# Evaluation of a Pre-Transplant Serum FTIR Rejection-Risk Score Across Time Horizons and Rejection Phenotype-Defined Endpoints in Kidney Transplantation

**DOI:** 10.3390/jpm16090485

**Published:** 2026-09-21

**Authors:** Luis Ramalhete, Rúben Araújo, Emanuel Vigia, Miguel Bigotte Vieira, Anibal Ferreira, Cecilia R. C. Calado

**Affiliations:** 1 Blood and Transplantation Center of Lisbon, Instituto Português do Sangue e da Transplantação, Alameda das Linhas de Torres, No. 117, 1769-001 Lisbon, Portugal; 2 NOVA Medical School, Faculdade de Ciências Médicas, Universidade NOVA de Lisboa, 1169-056 Lisbon, Portugal; 3 iNOVA4Health—Advancing Precision Medicine, Núcleo de Investigação em Doenças Renais, NOVA Medical School, Faculdade de Ciências Médicas, Universidade NOVA de Lisboa, 1169-056 Lisbon, Portugal; 4 LBS—Lisbon Business & Government School, Rua de São Bernardo, 34-A, 1200-427 Lisbon, Portugal; 5 Hepatobiliopancreatic and Transplantation Center, Curry Cabral Hospital, Unidade Local de Saúde de São José, R. da Beneficência 8, 1050-099 Lisbon, Portugal; 6 Centro Clínico Académico de Lisboa, Faculdade de Ciências Médicas, Universidade NOVA de Lisboa, 1169-056 Lisbon, Portugal; 7 Nephrology, Hospital Curry Cabral, Unidade Local de Saúde de São José, R. da Beneficência 8, 1050-099 Lisbon, Portugal; 8 ISEL—Instituto Superior de Engenharia de Lisboa, Instituto Politécnico de Lisboa, Rua Conselheiro Emídio Navarro 1, 1959-007 Lisbon, Portugal; 9 Institute for Bioengineering and Biosciences (iBB), The Associate Laboratory Institute for Health and Bioeconomy-i4HB, Instituto Superior Técnico (IST), Universidade de Lisboa (UL), Av. Rovisco Pais, 1049-001 Lisbon, Portugal

**Keywords:** fourier-transform infrared spectroscopy, kidney transplantation, rejection, time-to-rejection, risk phenotyping, machine learning, humoral rejection, cellular rejection, survival analysis, decision curve analysis

## Abstract

**Background:** Pre-transplant serum Fourier-transform infrared spectroscopy (FTIRS) has identified an internally validated biochemical signal associated with subsequent biopsy-proven kidney allograft rejection. Whether this signal retains discrimination across post-transplant time horizons and rejection-phenotype-defined endpoints remains unclear. **Methods:** This internally conducted extension study reused the same single-center cohort of 79 kidney transplant recipients (40 with biopsy-proven rejection; 39 with five-year rejection-free follow-up). The primary analysis did not retrain the original model; the fixed nested out-of-fold FTIR-derived score was evaluated at cumulative rejection horizons of 30, 90, 180, 365, 730, 1095, and 1825 days. Discrimination was assessed using AUCs with bootstrap confidence intervals and permutation testing. Cox regression, C-index, Kaplan–Meier analysis, exploratory decision curve analysis, and exploratory endpoint-specific models were also performed. **Results:** The fixed score retained discrimination across horizons, with AUCs of 0.780, 0.829, 0.845, and 0.860 at 30, 90, 180, and 365 days, respectively, and 0.850 at 1825 days. In Cox analysis, the FTIRS score was associated with rejection risk (HR per SD 1.95, 95% CI 1.40–2.73; *p* = 0.00009; C-index 0.709). Endpoint-specific models showed variable discrimination but involved small subgroups. **Conclusions:** The fixed pre-transplant FTIRS score retained discriminatory value across cumulative rejection-time horizons, with numerically highest discrimination within the first year. Endpoint-specific findings were hypothesis-generating and did not establish time- or phenotype-specific spectral signatures. Independent external validation is required.

## 1. Introduction

Kidney transplantation offers a survival advantage over remaining on long-term dialysis for appropriately selected patients with kidney failure, but durable allograft survival remains limited by immune-mediated injury and rejection-related graft loss [1,2]. Importantly, kidney allograft rejection is not a single biological entity. It comprises temporally and mechanistically heterogeneous processes, including antibody-mediated rejection (ABMR), T cell-mediated rejection (TCMR), mixed phenotypes, and microvascular inflammation. The Banff classification has progressively refined these diagnostic categories by integrating histological, serological, and molecular information, and recent outcome data have reinforced the prognostic relevance of microvascular inflammation and antibody-mediated injury [3,4].

Risk assessment in kidney transplantation currently relies on complementary clinical, immunological, histopathological, and molecular layers. Pre-transplant immunological evaluation, including sensitization to human leukocyte antigens (HLA) and donor-specific anti-HLA antibodies (DSA), complement-binding antibody assays, and HLA compatibility, remains central to alloimmune risk stratification [5]. After transplantation, graft-function monitoring and kidney allograft biopsy remain core components of rejection diagnosis, while molecular biopsy platforms, donor-derived cell-free DNA, urinary-cell messenger RNA (mRNA) profiles, chemokine signatures, and extracellular vesicle-based approaches have expanded the capacity to detect graft injury or intragraft immune activation [6,7,8,9,10]. However, these tools assess different biological windows. Some estimate pre-existing alloimmune risk, whereas others mainly detect injury or inflammation after the graft has already been exposed to the recipient immune environment. This distinction creates a rationale for complementary pre-transplant phenotyping strategies capable of capturing systemic biochemical states before graft implantation.

Fourier-transform infrared spectroscopy (FTIRS) is a label-free vibrational spectroscopy technique that can generate high-dimensional biochemical fingerprints from biological samples. In biofluids, FTIR spectra contain overlapping contributions broadly related to proteins, lipids, carbohydrates, nucleic acids, and other molecular structures, and therefore require multivariate analysis rather than interpretation as isolated molecular measurements [11,12,13,14,15,16]. FTIR-based biofluid analysis has been researched as a rapid and scalable approach for clinical phenotyping, particularly when combined with appropriate preprocessing, feature selection, and machine-learning workflows [12,13,14,16]. In kidney transplantation, our previous work explored serum FTIRS as a complementary strategy for rejection-related phenotyping [13,14]. More recently, a pre-transplant FTIRS study using nested feature selection and leakage-aware machine learning identified an internally validated spectral signal associated with subsequent biopsy-proven rejection [7]. That work supported the existence of a global pre-transplant rejection-risk signal, but it did not examine whether the discriminatory information carried by the fixed score persisted across clinically relevant post-transplant time horizons or whether exploratory rejection-phenotype-defined endpoints showed different levels of discrimination relative to rejection-free controls.

The present study was designed as an internal temporal and rejection-phenotype-defined extension of the previously established pre-transplant FTIRS risk-phenotyping framework. The primary aim was not to replace, re-estimate, or optimize the original global rejection classifier, but to determine whether its fixed out-of-fold score retained discriminatory value across progressively expanding cumulative rejection-time horizons. This analysis provides information that was not addressed in the original binary rejection-versus-no-rejection study: whether the same locked score remains informative for rejection occurring within different clinically relevant follow-up windows and whether it is associated with observed time to first rejection. Secondary analyses explored rejection-phenotype-defined and interval-specific rejection endpoints relative to five-year rejection-free controls. These exploratory analyses were intended to generate hypotheses regarding potential heterogeneity in the pre-transplant FTIRS signal, not to establish independent time-specific or rejection-phenotype-specific spectral signatures.

## 2. Materials and Methods

### 2.1. Study Design, Setting, and Analytical Framework

This single-center observational study was conducted at *Unidade Local de Saúde de São José*, *Hospital Curry Cabral*, Lisbon, Portugal. The study was designed as an internal temporal and rejection-phenotype-defined extension of a previously published pre-transplant serum FTIRS rejection-risk study using the same analytical cohort [7]. The study involved pre-transplant serum samples used for research between August 2022 and April 2026. The present analysis was not designed to replace, re-estimate, or optimize the previously reported global FTIRS rejection classifier. Instead, the primary objective was to evaluate whether the fixed nested out-of-fold FTIRS-derived risk score from the previous model retained discriminatory value across clinically relevant time-to-rejection horizons.

The analytical framework was structured to reduce model-shopping and information leakage, which are recognized concerns in high-dimensional small-sample prediction studies [17,18,19,20,21,22]. The primary analysis used the previously generated out-of-fold FTIRS probability score without retraining the original model, without reselecting spectral variables, and without changing the preprocessing pipeline. Cumulative time-horizon analyses, survival analyses, and decision curve analysis were therefore performed using this fixed score. Additional target-specific temporal and rejection-phenotype-defined models were considered secondary and exploratory.

Reporting followed the STROBE statement for observational studies and the TRIPOD+AI statement for prediction-model reporting [23,24,25].

### 2.2. Study Cohort and Eligibility Criteria

Adult kidney transplant recipients with an available pre-transplant serum sample collected on the day of transplantation, before graft implantation, were eligible for inclusion. Patients younger than 18 years and patients with human immunodeficiency virus (HIV) infection were excluded, according to the criteria used in the previous FTIRS rejection-risk study [7].

A total of 80 pre-transplant serum samples were initially evaluated. After FTIRS spectral quality control, one spectrum was excluded, resulting in a final analytical cohort of 79 recipients. The cohort included 40 recipients who developed biopsy-proven kidney allograft rejection during post-transplant follow-up and 39 recipients with five-year follow-up without biopsy-proven rejection. The five-year rejection-free group was used as the reference group for cumulative time-horizon analyses up to 1825 days.

### 2.3. Baseline Clinical and Immunological Variables

Baseline variables were extracted from clinical and laboratory records and included age at transplantation, sex, dialysis modality, retransplantation status, donor type, maximum complement-dependent cytotoxicity panel-reactive antibody value (PRA-CDC max), pre-transplant donor-specific anti-HLA antibodies (DSA), pre-transplant anti-HLA antibodies, and HLA-A, HLA-B, and HLA-DR mismatch burden.

Continuous variables are reported as median and interquartile range, and categorical variables as number and percentage. Table 1 reproduces the baseline cohort characteristics from the previous FTIRS rejection-risk article because the present study is an analytical extension of the same cohort [7]. Between-group *p*-values are shown descriptively and were not used for variable selection or for defining the FTIRS-based analytical strategy.

### 2.4. Serum Collection, FTIRS Acquisition, and Spectral Regions

Pre-transplant serum samples were collected on the day of kidney transplantation, before graft implantation. FTIRS acquisition and preprocessing were performed as previously described [7]. Briefly, serum samples were analysed as dried films after dilution in Milli-Q water. Spectra were acquired in transmission mode using a Vertex 70 FTIR spectrometer (Bruker, Mannheim, Germany) equipped with an HTS-XT accessory (Bruker, Ettlingen, Germany). Spectral acquisition covered the 400–4000 cm^−1^ range at 2 cm^−1^ resolution.

For the FTIRS-derived risk score used in the primary analysis, the predefined spectral windows were the biochemical fingerprint region from 600 to 1900 cm^−1^ and the high-wavenumber region from 2800 to 3400 cm^−1^ [7]. These regions were retained unchanged from the previous study to avoid re-optimizing spectral-region selection in the current temporal extension analysis. The use of predefined spectral regions and stable preprocessing is particularly important in FTIRS biofluid studies, where pre-analytical handling, spectral preprocessing, and high-dimensional model selection can strongly influence downstream classification performance [8,9,16,26,27,28].

No additional spectral quality-control exclusions were applied in the present extension. Horizon-specific analyses excluded only recipients whose rejection occurred after the corresponding cumulative cutoff.

### 2.5. Fixed FTIRS-Derived Risk Score

The primary exposure was the fixed out-of-fold probability score generated by the previously published nested FTIRS model [7]. The original pipeline used the combined 600–1900 and 2800–3400 cm^−1^ spectral windows, second-derivative transformation followed by normalization, FCBF feature selection nested within LOOCV, and a Naïve Bayes classifier [7,17]. As feature selection and model fitting were nested within each training fold, each recipient was predicted without contributing to feature selection or classifier training in that fold, reducing information leakage and optimistic performance estimation [18].

The present study deliberately used these fixed out-of-fold probabilities without retraining, recalibrating, or reselecting spectral features for the primary temporal analysis. This design was selected to test whether the previously identified global pre-transplant FTIR rejection-risk signal contained temporal information while reducing the risk of data-driven model selection and optimistic performance estimation [19,20,21].

### 2.6. Rejection Outcomes and Time-to-Rejection Definitions

The primary clinical outcome was biopsy-proven kidney allograft rejection after transplantation. Rejection time was defined as the interval, in days, between transplantation/pre-transplant serum sampling and the first documented biopsy-proven rejection episode.

Rejection phenotype was classified descriptively as humoral rejection, cellular rejection, or mixed rejection according to the diagnostic classification recorded in the clinical/pathology report. Mixed rejection was defined as the coexistence of humoral and cellular rejection features. Subtype analyses were considered exploratory due to the limited number of events within each rejection phenotype.

### 2.7. Cumulative Time-Horizon Analysis

The primary temporal analysis evaluated the fixed FTIRS-derived score across progressively expanding cumulative rejection-time horizons. The main prespecified horizons were 30, 90, 180, 365, 730, 1095, and 1825 days after transplantation. These cutoffs were selected to represent very early rejection, early rejection, intermediate rejection, first-year rejection, two-year rejection, three-year rejection, and five-year rejection.

For each time horizon, recipients with biopsy-proven rejection occurring on or before the cutoff were labelled positive. Recipients with five-year rejection-free follow-up were labelled negative. Recipients who developed rejection after the cutoff were excluded from that horizon-specific binary analysis, for they could not be considered true no-rejection controls for that time horizon.

The maximum observed rejection time of 2291 days was reported only as a contextual global endpoint corresponding to the original binary rejection-versus-no-rejection analysis [7], not as a primary cumulative time horizon, because the no-rejection reference group was defined by five-year rejection-free follow-up.

### 2.8. Survival Analysis

Time-to-event analysis was performed to assess the association between the fixed FTIRS-derived score and rejection-free survival. Recipients who developed biopsy-proven rejection were considered to have experienced the event at the time of first rejection. Recipients without biopsy-proven rejection were censored at five years, corresponding to 1825 days of rejection-free follow-up. Death and graft loss were not modelled as competing events in the present analysis. Accordingly, the Cox model was used to evaluate the association between the FTIRS-derived score and observed time to first biopsy-proven rejection within the analytical framework of this temporal extension, rather than to estimate the cumulative incidence of rejection in the presence of competing risks. This limitation was considered explicitly when interpreting the survival results.

Cox proportional hazards regression was used to estimate hazard ratios (HRs) per one standard deviation (SD) increase in score, with 95% confidence intervals (CIs) and *p*-values. Model discrimination in survival analysis was summarized using the concordance index (C-index).

Clinical-only and clinical-plus-FTIRS scores were evaluated as contextual comparator analyses. The clinical-only score was derived from prespecified baseline variables available in the cohort, including age, sex, retransplantation status, donor type, PRA-CDC max, pre-transplant DSA status, and HLA mismatch burden. The clinical-plus-FTIR score combined the fixed FTIR-derived score with the same baseline clinical and immunological variables. These comparator analyses were exploratory and were not used to modify, recalibrate, or select the primary FTIRS score. The interpretation of these analyses followed established recommendations that prediction models require appropriate internal, internal-external, and external validation before claims of clinical generalizability [19].

### 2.9. Discrimination, Confidence Intervals, and Permutation Testing

Discrimination of the fixed FTIRS-derived score for each cumulative time horizon was assessed using the area under the receiver operating characteristic curve (AUC). AUC confidence intervals were estimated using 5000 participant-level non-parametric bootstrap resamples, and 95% confidence intervals were calculated using the percentile method. Bootstrap resamples containing only one outcome class were excluded from confidence interval estimation.

Permutation testing was used to assess whether the observed AUC exceeded chance expectation. For each cumulative time horizon, rejection labels were randomly permuted while keeping the FTIRS-derived score fixed, and the AUC was recalculated after each permutation. A total of 5000 label permutations were performed for each time horizon. Empirical *p*-values were calculated as: *p* = (number of permuted AUCs ≥ observed AUC + 1)/(number of permutations + 1).

This correction was used to avoid reporting zero *p*-values when no permuted AUC equaled or exceeded the observed AUC. With 5000 permutations, the minimum reportable empirical *p*-value was therefore (0 + 1)/(5000 + 1) = 0.0002 after rounding. This minimum value could occur for more than one endpoint whenever none of the 5000 permuted AUCs reached the corresponding observed AUC. In the present analysis, this occurred for the 90-, 180-, 365-, 730-, 1095-, and 1825-day cumulative horizons, as well as for the contextual global endpoint at 2291 days. This permutation strategy tested whether the fixed FTIRS-derived score was associated with temporal rejection labels beyond random label assignment, without retraining or modifying the original FTIRS model.

### 2.10. Decision Curve Analysis

Decision curve analysis (DCA) was performed as an exploratory decision-analytic assessment of the fixed FTIRS-derived score [29]. Net benefit was calculated across a clinically plausible range of threshold probabilities and compared with treat-all and treat-none strategies. DCA was performed for selected cumulative rejection horizons, including 90, 180, 365, and 1825 days.

Since the FTIRS-derived score has not been externally calibrated, the threshold probabilities used in the present DCA cannot be assumed to correspond to calibrated absolute rejection risks. Miscalibration may bias estimated net benefit in either direction, resulting in either overestimation or underestimation of apparent clinical benefit depending on the direction and magnitude of calibration error at the relevant thresholds. In addition, the approximately balanced analytical cohort was assembled for risk-phenotyping purposes and should not be assumed to reflect rejection prevalence in an unselected kidney transplant population. Accordingly, the DCA was included solely as an exploratory graphical analysis and cannot currently establish clinical benefit, support clinical decision-making, or demonstrate implementation readiness. Formal assessment of clinical utility will require external validation and calibration in a representative target population before decision-curve analysis can be interpreted clinically [20,30,31].

### 2.11. Exploratory Target-Specific Temporal and Rejection-Phenotype-Defined Models

Exploratory target-specific models were trained for selected temporal and rejection-phenotype-defined endpoints to assess variation in discrimination relative to the five-year rejection-free reference group. These analyses were secondary, hypothesis-generating, and were not intended to provide stable estimates of endpoint-specific predictive performance.

The selected exploratory endpoints included first-year rejection, late rejection beyond 365 days, humoral rejection, cellular rejection, first-year humoral rejection, and first-year cellular rejection, each compared with the five-year no-rejection reference group. Additional discrete interval analyses were performed for selected intervals with sufficient event counts, including rejection occurring within 0–30 days, 31–90 days, 31–180 days, and beyond 365 days. Subgroups with extremely low event counts were not generally used as standalone target-specific modelling endpoints. Specifically, cellular rejection within 0–30 days (*n* = 3) and mixed rejection (*n* = 5 overall) were not modelled separately. Some exploratory interval-specific analyses nevertheless involved 10 or fewer rejection events, including humoral rejection within 0–30 days (*n* = 8) and cellular rejection between 31 and 180 days (*n* = 6). These analyses were retained solely for hypothesis generation and were not considered estimates of stable or clinically actionable predictive performance.

To limit overfitting, exploratory target-specific models were restricted to the same prespecified FTIRS windows used in the primary score analysis, namely 600–1900 and 2800–3400 cm^−1^. Feature selection was performed using FCBF nested within each LOOCV training fold [17,18]. Classifiers were restricted to a small prespecified set of relatively simple models suitable for high-dimensional, small-sample spectral data, including Naïve Bayes, penalized logistic regression, and linear support vector machine models. More flexible models, when tested, were interpreted as supplementary and hypothesis-generating only.

Performance metrics for exploratory models included AUC, accuracy, sensitivity, specificity, balanced accuracy, F1-score, and feature-selection frequency across LOOCV folds. Considering the relatively small subgroup sizes, these analyses were not interpreted as clinically actionable classifiers. This conservative interpretation is consistent with methodological concerns regarding feature selection, small sample size, calibration, and validation in clinical prediction modelling [18,19,20,30,32].

### 2.12. Statistical Software and Reproducibility

Baseline demographic, clinical, and immunological variables were analyzed to characterize the study cohort and to assess potential between-group differences. Statistical analyses of clinical and demographic variables were performed using GraphPad Prism version 8.2.0 (GraphPad Software, San Diego, CA, USA). Continuous variables were expressed as median and interquartile range and compared using the Mann–Whitney U test. Binary categorical variables were expressed as number and percentage and compared using Fisher’s exact test. Dialysis modality, as a categorical variable with more than two levels, was compared using the Fisher–Freeman–Halton exact test. A two-sided *p*-value < 0.05 was considered statistically significant. Machine-learning analyses were performed using Python 3.13.5. The analytical environment included Python and different modules, e.g., NumPy, pandas, scikit-learn, SciPy, statsmodels, and matplotlib.

For cumulative and rejection-phenotype-specific endpoints, the exploratory pipeline reported in Table S1 was selected according to the highest balanced accuracy. For discrete interval-specific endpoints, the pipeline reported in Table S3 was selected according to the highest AUC within the limited prespecified model set. These analyses addressed different exploratory modelling frameworks and were not compared as competing final models.

### 2.13. Ethics

The study was conducted in accordance with the Declaration of Helsinki and was approved by the ULS São José Ethics Committee (approvals No. 1215/2022, 4 July 2022). Written informed consent for the research use of clinical data and stored samples was obtained from recipients in accordance with institutional policy and applicable regulations.

## 3. Results

### 3.1. Cohort Characteristics and Rejection Timing

The final analytical cohort included 79 kidney transplant recipients, of whom 40 developed biopsy-proven rejection and 39 had five-year follow-up without biopsy-proven rejection. Since the present study is an internal temporal and rejection-phenotype-defined extension of the previously published pre-transplant FTIRS rejection-risk study, Table 1 reproduces the baseline characteristics of the same analytical cohort for transparency and comparability.

Among the 40 rejection episodes, 22 were classified as humoral rejection, 13 as cellular rejection, and 5 as mixed rejection. Rejection timing was heterogeneous. Twelve rejection episodes occurred within 30 days, 20 within 90 days, 22 within 180 days, and 26 within the first year after transplantation. Fourteen rejection episodes occurred past one year. The complete temporal distribution of rejection events and immunophenotypes is provided in Table S4 and Supplementary Figure S1.

The distribution of rejection phenotypes varied across time. Very early rejection within 30 days included 8 humoral, 3 cellular, and 1 mixed episode. By one year, the cumulative rejection endpoint included 12 humoral, 10 cellular, and 4 mixed episodes. This indicates that the first-year rejection endpoint was not driven exclusively by a single rejection phenotype.

### 3.2. Fixed Published FTIRS Score Across Cumulative Time-to-Rejection Horizons

**Figure 1:**
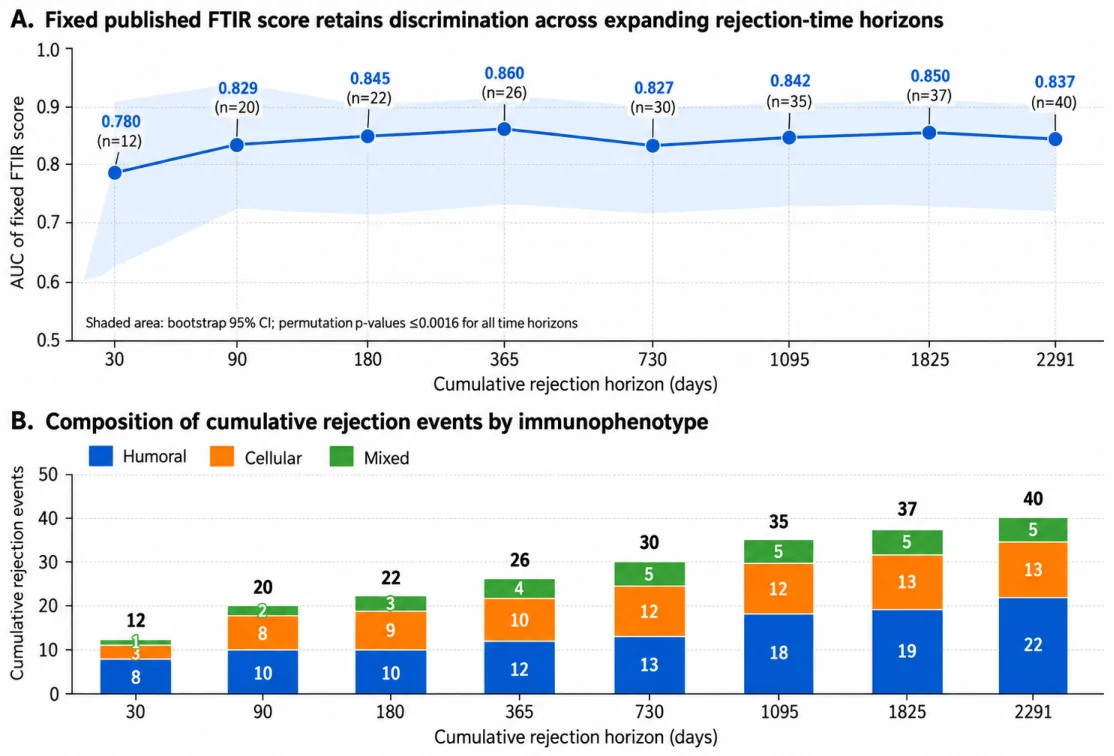
Discrimination of the fixed pre-transplant FTIR-derived rejection-risk score across cumulative rejection-time horizons. (**A**) Discriminative performance of the fixed published FTIR-derived score across progressively expanding cumulative rejection-time horizons. The shaded area represents bootstrap 95% confidence intervals. The 2291-day point represents the original global rejection endpoint and is shown as contextual reference. (**B**) Cumulative composition of rejection events by immunophenotype across time horizons. FTIRS, Fourier-transform infrared spectroscopy; AUC, area under the receiver operating characteristic curve.

The fixed pre-transplant FTIRS-derived score retained discriminatory ability across progressively expanding cumulative rejection-time horizons without retraining, recalibration, or feature reselection of the original model (Table 2 and Figure 1).

Discrimination was already evident for very early rejection within 30 days, with an AUC of 0.780 (95% CI 0.602–0.923; permutation *p* = 0.0016). The corresponding AUC estimates were 0.829 for rejection within 90 days, 0.845 within 180 days, and 0.860 within 365 days.

The first-year endpoint showed the numerically highest discrimination among the main cumulative horizons, with AUC 0.860 (95% CI 0.756–0.945; permutation *p* = 0.0002). Discrimination remained stable at later follow-up horizons, with AUC 0.827 at 730 days, 0.842 at 1095 days, and 0.850 at 1825 days.

The original global rejection endpoint, including all rejection events up to the maximum observed rejection time of 2291 days, showed AUC 0.837 (95% CI 0.741–0.920; permutation *p* = 0.0002). This value is reported as a contextual reference to the previously published global rejection-versus-no-rejection analysis, rather than as a primary five-year time-horizon endpoint.

Overall, these findings indicate that the previously identified pre-transplant FTIR rejection-risk signal was not limited to a binary rejection-versus-no-rejection endpoint. Instead, the fixed score retained discrimination across cumulative rejection-time horizons, with the numerically highest AUC observed for biopsy-proven rejection occurring within the first year after transplantation. Since the horizon-specific AUC estimates were correlated, their confidence intervals overlapped, and no formal comparisons between horizons were performed, these findings should not be interpreted as evidence of differential predictive performance across time horizons.

### 3.3. Time-to-Event Analysis

In Cox proportional hazards analysis, the FTIRS-only score was significantly associated with time to biopsy-proven rejection (Table 3). Each one standard deviation increase in the FTIRS-derived score was associated with a hazard ratio of 1.95 for rejection (95% CI 1.40–2.73; *p* = 0.00009), with a C-index of 0.709.

The clinical-only score showed limited discrimination in this cohort, with HR 1.16 per one standard deviation increase (95% CI 0.86–1.57; *p* = 0.327) and C-index 0.513. The clinical-plus-FTIRS score remained associated with rejection risk, with HR 1.73 (95% CI 1.22–2.43; *p* = 0.0018) and C-index 0.616, compared with 0.709 for the FTIRS-only score. No formal statistical comparison between C-indices was performed; therefore, these differences should be interpreted descriptively and do not establish superiority or incremental clinical value of FTIRS. These comparator analyses were exploratory. A graphical comparison of the hazard ratios and discrimination estimates for the three scores is provided in Figure S2.

**Figure 2:**
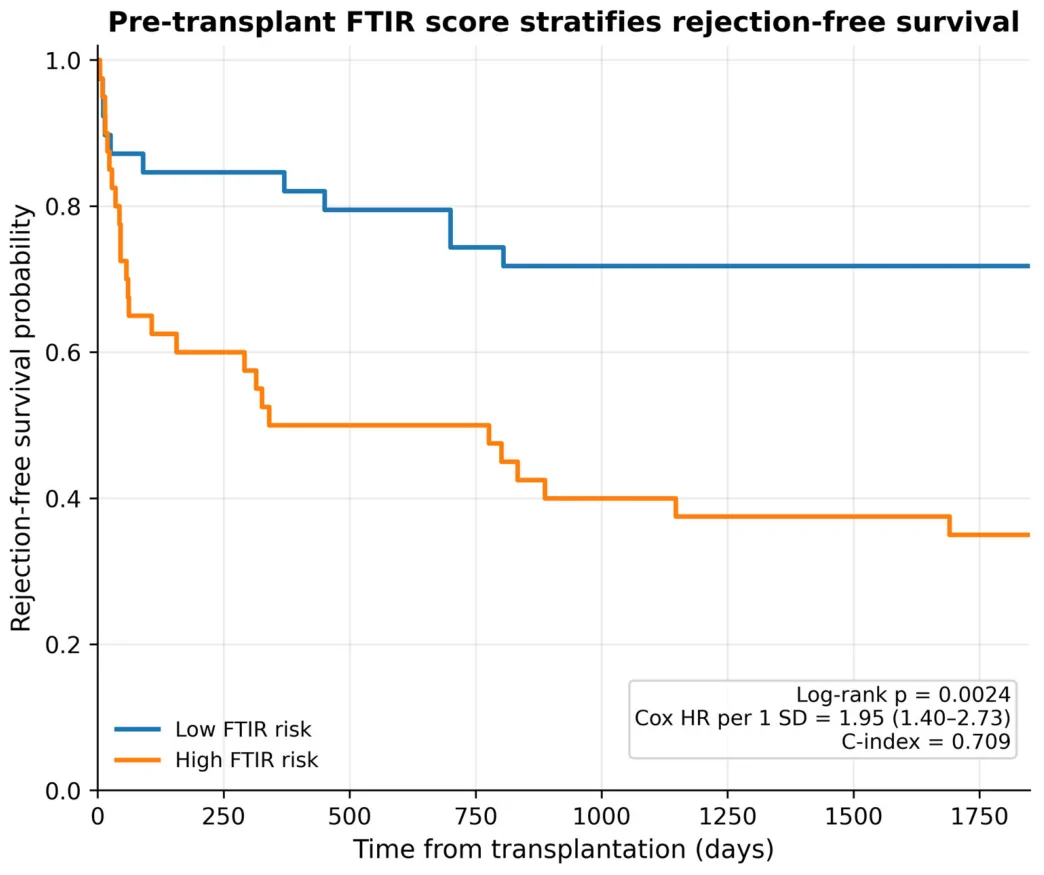
Rejection–free survival stratified by the fixed pre-transplant FTIRS-derived score. Kaplan–Meier curves show rejection-free survival after stratification by the median FTIRS-derived score. Recipients with higher FTIRS scores had lower rejection-free survival than recipients with lower FTIRS scores. The log-rank test was used to compare survival distributions. FTIRS, Fourier-transform infrared spectroscopy.

Kaplan–Meier analysis stratified by the median FTIRS-derived score showed lower rejection-free survival among recipients with higher FTIRS scores (log-rank *p* = 0.0024; Figure 2). These results support an association between the fixed pre-transplant FTIRS-derived score and observed rejection-free survival in this cohort, while recognizing that the survival analysis was internally derived, did not account for competing risks, and requires external confirmation.

### 3.4. Exploratory Decision Curve Analysis

**Figure 3:**
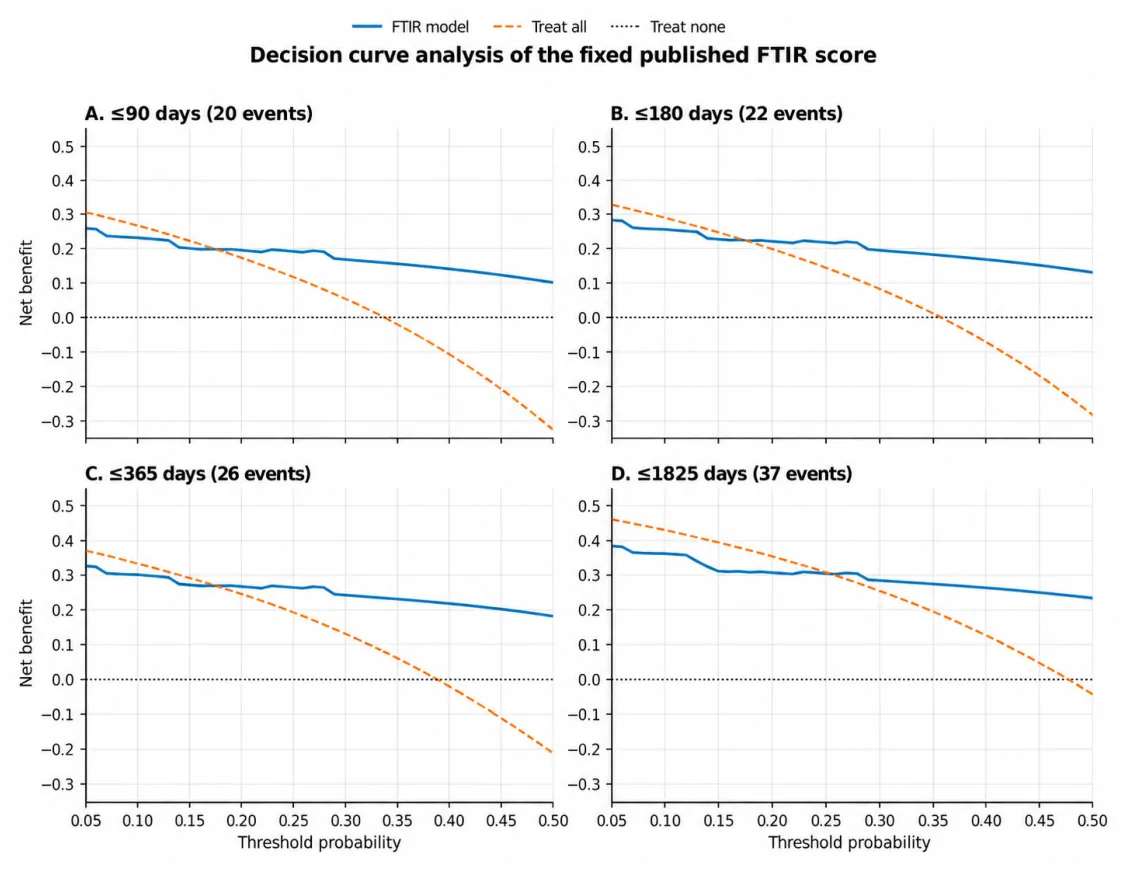
Exploratory decision curve analysis of the fixed pre-transplant FTIRS-derived score. Decision curve analysis was performed for rejection within (**A**) 90, (**B**) 180, (**C**) 365, and (**D**) 1825 days. Net benefit of the FTIRS-derived score was compared with treat-all and treat-none strategies across threshold probabilities. The number of biopsy-proven rejection events included in each horizon is shown directly in the corresponding panel. As the score has not been externally calibrated, the threshold probabilities cannot be assumed to represent calibrated absolute rejection risks, and miscalibration may bias the estimated net benefit either upward or downward. In addition, the nearly balanced analytical cohort should not be assumed to reflect rejection prevalence in an unselected transplant population. The curves should therefore be interpreted solely as exploratory and do not demonstrate clinical benefit.

Decision curve analysis showed graphical separation of the fixed FTIRS-derived score from the treat-all and treat-none strategies across parts of the evaluated threshold range (Figure 3). However, because the score has not been externally calibrated, these threshold probabilities cannot be interpreted as validated absolute rejection-risk thresholds, and the direction of any resulting net-benefit bias is unknown. The DCA is therefore presented solely as an exploratory analysis and does not demonstrate clinical benefit or support clinical decision-making.

### 3.5. Exploratory Target-Specific Temporal and Rejection-Phenotype-Defined Models

Exploratory target-specific models showed variable discrimination across selected temporal and rejection-phenotype-defined endpoints when each endpoint was compared separately with the five-year rejection-free reference group. These analyses were considered preliminary because they involved smaller endpoint-specific subgroups and additional model fitting beyond the fixed primary score analysis, and therefore require confirmation in larger independent cohorts. An overview of the principal target-specific results is shown in Figure S3, while detailed performance and analytical specifications of the exploratory temporal and rejection-phenotype-specific models are provided in Table S1. Across the nine exploratory models summarized in Table S1, endpoint-specific event counts ranged from 10 to 26, with 39 five-year rejection-free controls in each comparison. The models were generally sparse, with a median of 1–6 selected spectral features across LOOCV folds; eight of the nine models had a median of three or fewer selected features.

For first-year rejection versus five-year no rejection, the best-balanced target-specific model achieved AUC 0.815, sensitivity 0.769, specificity 0.846, and balanced accuracy 0.808. Late rejection beyond 365 days achieved AUC 0.795 and balanced accuracy 0.813.

Some cellular rejection analyses yielded numerically high discrimination, but these estimates were based on small event counts and should not be interpreted as evidence of superior or stable performance for cellular rejection. Cellular rejection within the first year achieved AUC 0.885, sensitivity 0.900, specificity 0.769, and balanced accuracy 0.835. Cellular rejection occurring between 31 and 180 days showed AUC 0.953, but this analysis included only six positive cases and should therefore be interpreted with particular caution.

Humoral rejection endpoints showed more moderate discrimination. Humoral rejection versus five-year no rejection achieved AUC 0.784, while late humoral rejection beyond 365 days achieved AUC 0.813. Mixed rejection was not modelled as a target-specific endpoint because only five mixed rejection episodes were available.

Permutation-based screening of the out-of-fold target-specific scores is reported in Table S2. Results from the discrete interval-specific models, including rejection within 0–30 days, 31–90 days, 31–180 days, and beyond 365 days, are provided in Table S3. The underlying distribution of rejection events by interval and immunophenotype is presented in Table S4 and Figure S1. The discrete interval-specific analyses reported in Table S3 were based on still smaller subgroups: event counts ranged from 6 to 14, and five of the seven models included 10 or fewer rejection events. Accordingly, their performance estimates should be regarded as particularly unstable.

Taken together, these exploratory analyses show that endpoint-specific models were able to discriminate selected temporal and rejection-phenotype-defined endpoints from five-year rejection-free controls within this cohort. Because several analyses involved very small event counts and no direct comparisons were performed between rejection phenotypes or between early and late rejection, these findings do not establish time-specific or rejection-phenotype-specific spectral signatures and should be regarded as hypothesis-generating.

## 4. Discussion

This study evaluated whether a previously developed pre-transplant FTIRS-derived rejection-risk score remained informative across clinically relevant post-transplant time horizons. The fixed out-of-fold score, retained without retraining, recalibration, or feature reselection, discriminated rejection across cumulative horizons from 30 to 1825 days and was associated with observed time to first rejection. Because the same single-center cohort was used, these findings represent an internal temporal extension rather than independent validation. The additional information lies in showing that the previously identified global signal remains associated with rejection across different follow-up windows. This persistence of discrimination does not establish distinct time-specific biological signatures, and the exploratory rejection-phenotype-defined models do not directly discriminate between rejection phenotypes.

The cumulative-horizon results indicate that the discriminatory performance of the pre-transplant score varied according to the time window considered, but they do not demonstrate prediction of the exact timing of rejection. For each prespecified cutoff, recipients who experienced rejection on or before that time point were compared with recipients who remained rejection-free for five years, while recipients who developed rejection after the cutoff were excluded from that specific analysis. These comparisons therefore represent cumulative case–control analyses rather than censoring-adjusted time-dependent ROC analyses. Because the same rejection-free reference group was used at each cutoff and progressively more rejection cases were included as the time window increased, the resulting AUC estimates were not independent. Although the AUC increased from 0.780 at 30 days to 0.860 at 365 days, the confidence intervals overlapped and no formal paired comparisons between time points were performed. The first-year result should therefore be described as the numerically highest discrimination among the prespecified time horizons, rather than as evidence of statistically superior performance at that time point.

A further consideration is that the fixed score was originally optimized to distinguish any subsequent rejection from no rejection, rather than to identify early or late events separately [7]. Its performance across cumulative horizons may therefore reflect persistence of the previously identified global rejection-risk signal among recipients who rejected by each cutoff, rather than a distinct biological signature specific to each time interval. The stability of discrimination through five years indicates that the pre-transplant signal was not confined to very early rejection. However, evidence for genuinely distinct temporal phenotypes depends primarily on the exploratory target-specific models and will require independent validation.

The biological heterogeneity of kidney allograft rejection provides an appropriate context for examining temporal patterns. Contemporary Banff frameworks recognize ABMR, TCMR, mixed phenotypes, and different forms of microvascular inflammation as related but non-equivalent manifestations of allograft injury [3,4]. Microvascular inflammation and antibody-mediated phenotypes are associated with clinically meaningful differences in graft outcome [3]. ABMR itself encompasses heterogeneous clinical and molecular presentations, including early inflammatory, later remodeling-dominant, and combined phenotypes, while subclinical ABMR and TCMR detected at one year represent distinct prognostic states [33,34,35,36,37]. In the present cohort, both humoral and cellular rejection contributed to the first-year endpoint, whereas humoral rejection represented a substantial proportion of events occurring beyond one year. This distribution reinforces the relevance of examining rejection beyond a single binary endpoint, but it does not establish that the fixed FTIRS score preferentially identifies one rejection mechanism or that changes in its discrimination are caused by the evolving immunophenotypic composition.

FTIR spectroscopy must also be interpreted according to the biological information it measures. A serum FTIR spectrum is a composite biochemical fingerprint generated by overlapping vibrational contributions from proteins, lipids, carbohydrates, nucleic-acid-related structures, and other circulating components [8,9,12,16]. It does not directly quantify a single immune mediator, antibody, metabolite, or molecular pathway. The observed signal should therefore be interpreted as a composite and biologically non-specific biochemical pattern. Any relationship with immune activation, inflammation, metabolic stress, oxidative processes, lipid composition, protein structure, or other molecular pathways remains hypothetical and cannot be inferred from the FTIR data alone. In complex biofluids, individual wavenumbers cannot be assigned unequivocally to specific molecules or causal pathways solely from their spectral position. Orthogonal proteomic, metabolomic, lipidomic, and immunological analyses will therefore be required to establish the molecular determinants of the FTIR-derived score [8,9,12,16,28].

The pre-transplant sampling window differentiates this approach from most established and emerging rejection biomarkers. Anti-HLA sensitization, DSA, complement-binding capacity, and donor–recipient compatibility characterize important components of baseline alloimmune risk [5,33]. By contrast, donor-derived cell-free DNA, urinary-cell transcript profiles, chemokine signatures, and molecular biopsy platforms predominantly identify graft injury or intragraft immune activation after transplantation [1,6,38,39,40]. The FTIRS score was obtained before graft implantation and may therefore reflect a recipient-level biochemical phenotype recorded before the graft is exposed to the post-transplant immune and metabolic environment. Its most plausible potential role would be as an additional systems-level layer within multimodal pre-transplant risk assessment, rather than as a substitute for DSA testing, graft-function monitoring, post-transplant biomarkers, or biopsy-based diagnosis.

The time-to-event analyses provided evidence complementary to the cumulative binary endpoints. Each standard deviation increase in the FTIRS-derived score was associated with a hazard ratio of 1.95 for first biopsy-proven rejection, and the FTIRS-only score achieved a C-index of 0.709. Kaplan–Meier curves also showed lower rejection-free survival among recipients with higher scores. These results indicate an association between the continuous FTIRS score and the occurrence of rejection during follow-up. However, a C-index of 0.709 represents moderate rather than definitive individual-level discrimination. Furthermore, dichotomization at the median was used for graphical presentation and does not define a clinically validated risk threshold. The continuous-score Cox model should therefore remain the principal survival result.

The clinical model analyses require similarly cautious interpretation. The clinical-only score showed limited discrimination, whereas the FTIRS-only score achieved a higher C-index in this cohort. This finding does not establish that FTIRS is superior to contemporary clinical or immunological assessment. The clinical reference model included only a restricted set of retrospectively available baseline variables and did not capture the full range of information used in transplant risk assessment, including detailed DSA characteristics, antibody strength or complement-binding capacity, donor quality, ischemia-related variables, immunosuppression exposure, adherence, post-transplant DSA evolution, infections, or longitudinal graft-function data. In addition, the small cohort limited the number of variables that could be incorporated without generating unstable estimates. A formal assessment of incremental value will require a prespecified comparison with a more comprehensive clinical reference model in a substantially larger cohort, together with evaluation of calibration, discrimination, and decision-analytic performance [19,20,30,32]. No formal statistical comparison of the C-indices was performed; therefore, the observed differences between the FTIR-only, clinical-only, and combined models should be interpreted descriptively rather than as evidence of model superiority. Accordingly, no claim of incremental clinical value of FTIRS over established clinical or immunological assessment is made in the present study.

The lower C-index observed for the clinical-plus-FTIRS model compared with the FTIR-only model should not be interpreted as evidence that clinical information reduces predictive performance. In a small cohort, adding several predictors can increase model instability and lead to poorer internal performance estimates without implying that the additional variables are clinically uninformative. The combined model was exploratory and was not developed or validated as a definitive multimodal prediction model. Larger studies with sufficient numbers of events will be needed to compare clinical-only, FTIR-only, and combined approaches using prespecified predictors and modelling strategies designed to limit overfitting [20,32].

Decision curve analysis showed graphical separation between the fixed FTIR score and treat-all or treat-none strategies across parts of the evaluated threshold range. However, these curves cannot currently be interpreted as evidence of clinical benefit. Decision curve analysis depends on risk estimates that are sufficiently calibrated for the threshold probabilities to have clinical meaning [30,31]. The present FTIRS-derived probabilities have not undergone external calibration, and miscalibration may bias estimated net benefit in either direction, either overestimating or underestimating apparent benefit depending on whether predicted risks are too high or too low at the relevant thresholds. In addition, the nearly balanced analytical cohort was assembled for risk-phenotyping analysis and should not be assumed to reflect rejection prevalence in an unselected transplant population. No clinical intervention was prespecified for recipients exceeding a particular FTIRS threshold. The present DCA should therefore be regarded solely as an exploratory graphical analysis and does not establish clinical utility or support clinical decision-making.

Future decision-curve analyses should first define the intended clinical decision. Potential applications could include intensified DSA surveillance, closer monitoring of graft function, earlier use of donor-derived cell-free DNA or other complementary biomarkers, or consideration of protocol biopsy in selected high-risk recipients. The model would then require recalibration in the target population, and net benefit should be assessed at threshold probabilities reflecting the clinical consequences of the proposed action [30,31,35,36].

The target-specific analyses explored variation in discrimination across selected temporal and rejection-phenotype-defined endpoints relative to rejection-free controls. Some models yielded numerically high AUC estimates, including cellular rejection within the first year and cellular rejection occurring between 31 and 180 days. These results are potentially informative for hypothesis generation, but they are also the least stable findings of the study. The cellular 31–180-day analysis included only six positive cases, and several other subtype-specific endpoints contained similarly small numbers of events. With event counts of this magnitude, classification of a single additional case could materially alter sensitivity and other performance estimates. The apparently high AUCs of these models should therefore be viewed as imprecise exploratory point estimates rather than evidence of reproducible endpoint-specific prediction. Multiple preprocessing approaches and classifiers were evaluated before the best-performing pipelines were selected. Nested feature selection within LOOCV reduced information leakage but could not eliminate the high variance, selection optimism, and limited calibration associated with very small event counts and multiple candidate models [17,18,19,20,32].

The permutation analyses of the exploratory models should also be interpreted differently from those applied to the fixed primary score. For these analyses, the already generated out-of-fold scores were evaluated after random reassignment of the outcome labels. The complete analytical pipeline, including preprocessing, feature selection, classifier selection, and model fitting, was not repeated within each permutation. Nevertheless, the results support the stability of the observed association between the fixed scores and the corresponding clinical endpoints under label randomization. They should therefore be viewed as complementary evidence supporting the exploratory findings rather than as full permutation validation of the entire model-development procedure. Confirmation in independent cohorts will be important to establish the reproducibility and generalizability of the endpoint-specific models.

The rejection-phenotype-defined analyses should not be interpreted as direct classification of rejection mechanism. The humoral and cellular models were separate rejection-versus-control comparisons, each using recipients who remained rejection-free for five years as the reference group. They were not designed as a direct humoral-versus-cellular, or ABMR-versus-TCMR, classification task among recipients who subsequently rejected. Similarly, the interval-specific models compared rejection occurring within a selected interval with five-year no rejection rather than directly discriminating early from late rejection. The findings therefore suggest phenotype- and interval-associated patterns relative to rejection-free controls, but they do not support prospective determination of the exact rejection phenotype or timing. Mixed rejection could not be modelled reliably because only five cases were available.

Key strengths include evaluation of a fixed out-of-fold score without re-optimization, use of a five-year rejection-free reference group, and complementary assessment using cumulative discrimination and time-to-event analyses. These design features reduce opportunities for additional optimism but do not substitute for independent validation.

This study has several important limitations. First, it was a single-center analysis based on the same 79-recipient cohort used to develop the original FTIRS model. The present work therefore provides a new temporal interpretation of internally generated predictions but does not constitute independent patient-level validation. Second, the fixed score was developed against global rejection status; consequently, the cumulative-horizon analyses cannot by themselves establish the existence of time-specific biological signatures. Third, the cumulative horizons were nested and shared both controls and an increasing proportion of rejection cases, resulting in correlated performance estimates; no formal pairwise comparison of AUCs between horizons was performed. Fourth, event counts were limited for several rejection phenotypes and discrete time intervals, and the selection of the best-performing exploratory pipelines among multiple preprocessing and classifier combinations may have introduced additional selection optimism. Fifth, rejection immunophenotypes were derived from the recorded clinical and pathology diagnoses and were not independently reclassified using a central molecular or study-specific pathology assessment. Sixth, the clinical comparator did not include the full range of baseline and longitudinal variables relevant to rejection, including detailed DSA characteristics, donor-related factors, post-transplant graft-function trajectories, immunosuppression exposure, treatment modifications, adherence, and infectious events. Seventh, death and graft loss were not modelled as competing events. The Cox analysis should therefore be interpreted as describing the association between the FTIRS-derived score and observed time to first biopsy-proven rejection within this cohort, rather than as estimating the cumulative incidence of rejection in the presence of competing risks. A future externally validated time-to-event analysis should explicitly account for competing events when complete event-specific follow-up is available. Eighth, the molecular basis of the FTIRS-derived signal remains unknown. Finally, external calibration, reproducibility across laboratories and instruments, and robustness to pre-analytical and analytical variability have not been established [8,11,16,19,30,32].

The next research step should be prospective multicenter external validation of the locked analytical protocol and model, without updating during the primary validation analysis. Performance should include discrimination, calibration, decision-analytic assessment, and comparison with a comprehensive clinical and immunological reference model [19,24,25,30,32,40]. Orthogonal proteomic, metabolomic, lipidomic, and immunological profiling would be required to investigate the biological correlates of the FTIRS-derived signal. Interventional evaluation should follow only after successful external validation.

Taken together, the findings provide convergent evidence that the locked pre-transplant FTIRS-derived score retains information across clinically relevant cumulative rejection horizons and is associated with rejection-free survival. The exploratory analyses do not establish interval-specific or rejection-phenotype-specific spectral signatures and should not be conflated with direct prediction of rejection timing or mechanism.

## 5. Conclusions

The fixed pre-transplant serum FTIRS-derived score retained discrimination across clinically relevant cumulative rejection-time horizons and was associated with observed time to first biopsy-proven rejection. The numerically highest AUC was observed for rejection within the first year; however, the overlapping confidence intervals and absence of formal comparisons between horizons do not support a claim of statistically superior performance at this time point or prediction of the exact timing of rejection. Exploratory endpoint-specific models showed variable discrimination relative to rejection-free controls, but these findings were based on small subgroups and do not establish time-specific or rejection-phenotype-specific spectral signatures. The present study should therefore be regarded as an internal temporal extension of the previously reported global FTIRS rejection-risk signal rather than an independent validation study. Prospective multicenter validation of the locked model, external assessment of calibration and clinical utility, comparison with comprehensive clinical and immunological reference models, and orthogonal molecular characterization are required before clinical application.

## Figures and Tables

**Table 1:** Baseline characteristics of the analytical cohort. Values are median (IQR) or *n* (%).

Variable	Overall Cohort (*n* = 79)	No Rejection (*n* = 39)	Rejection (*n* = 40)	*p*-Value
Age at transplantation, years	44.0 (35.5–51.5)	46.0 (38.0–59.0)	42.5 (34.8–49.0)	0.093
Male sex, *n* (%)	49 (62.0)	25 (64.1)	24 (60.0)	0.818
Dialysis modality, *n* (%)				0.759
Hemodialysis	64 (81.0)	31 (79.5)	33 (82.5)	
Peritoneal dialysis	10 (12.7)	6 (15.4)	4 (10.0)	
Preemptive transplantation	5 (6.3)	2 (5.1)	3 (7.5)	
Retransplantation, *n* (%)	11 (13.9)	4 (10.3)	7 (17.5)	0.518
Deceased donor, *n* (%)	72 (91.1)	36 (92.3)	36 (90.0)	1.000
PRA-CDC max, %	4.0 (0.0–43.0)	6.0 (0.0–49.0)	0.0 (0.0–28.0)	0.421
Pre-transplant donor-specific antibodies, *n* (%)	14 (17.7)	5 (12.8)	9 (22.5)	0.378
Pre-transplant anti-HLA antibodies, *n* (%)	54 (68.4)	28 (71.8)	26 (65.0)	0.630
Total number of HLA mismatches (A-B-DR)	4.0 (3.0–5.0)	4.0 (3.0–5.0)	4.0 (3.0–5.0)	0.613
HLA-A mismatches	1.0 (1.0–2.0)	1.0 (1.0–2.0)	1.0 (1.0–2.0)	0.570
HLA-B mismatches	2.0 (1.0–2.0)	2.0 (1.0–2.0)	2.0 (1.0–2.0)	0.921
HLA-DR mismatches	1.0 (1.0–2.0)	1.0 (1.0–2.0)	1.0 (1.0–2.0)	0.096

**Table 2:** Discrimination of the fixed published FTIRS-derived score across cumulative rejection-time horizons.

Endpoint/Horizon	*n*	Rejection Events	No-Rejection Controls	Humoral	Cellular	Mixed	AUC	95% CI	Permutation *p*
≤30 days	51	12	39	8	3	1	0.780	0.602–0.923	0.0016
≤90 days	59	20	39	10	8	2	0.829	0.702–0.936	0.0002
≤180 days	61	22	39	10	9	3	0.845	0.726–0.938	0.0002
≤365 days	65	26	39	12	10	4	0.860	0.756–0.945	0.0002
≤730 days	69	30	39	13	12	5	0.827	0.725–0.912	0.0002
≤1095 days	74	35	39	18	12	5	0.842	0.747–0.919	0.0002
≤1825 days	76	37	39	19	13	5	0.850	0.753–0.925	0.0002
Global endpoint up to 2291 days	79	40	39	22	13	5	0.837	0.741–0.920	0.0002

95% confidence intervals were estimated using 5000 participant-level non-parametric bootstrap resamples. Permutation *p*-values were calculated using 5000 label permutations. A value of *p* = 0.0002 represents the minimum empirical *p*-value obtainable after rounding when none of the 5000 permuted AUCs equaled or exceeded the observed AUC; this occurred for multiple horizons. The 2291-day row represents the original global rejection endpoint and is shown as a contextual reference.

**Table 3:** Cox proportional hazards and discrimination analysis for FTIRS-only, clinical-only, and clinical plus FTIRS scores.

Model	HR per 1 SD	95% CI	*p*-Value	C-Index
FTIR-only score	1.95	1.40–2.73	0.00009	0.709
Clinical-only score	1.16	0.86–1.57	0.327	0.513
Clinical-plus-FTIR score	1.73	1.22–2.43	0.0018	0.616

## Data Availability

The data presented in this study are available on request from the corresponding author due to privacy and ethical restrictions related to patient-level clinical and laboratory data.

## Supplementary Materials

The following supporting information can be downloaded at: https://www.mdpi.com/article/10.3390/jpm16090485/s1, Figure S1: Distribution of rejection immunophenotypes across discrete post-transplant time intervals; Figure S2: Comparison of the associations between the evaluated scores and biopsy-proven rejection.; Figure S3: Exploratory target-specific FTIR model performance across temporal and rejection-phenotype-defined endpoints; Table S1: Performance of exploratory temporal and rejection-phenotype-specific FTIRS models selected by balanced accuracy; Table S2: Permutation-based screening of selected out-of-fold scores from exploratory target-specific FTIR models. These analyses assessed whether the observed discrimination of the selected out-of-fold scores exceeded that expected after random reassignment of endpoint labels; they did not constitute complete model-retraining permutation tests; Table S3: Best-performing exploratory FTIRS models for discrete rejection-time intervals. Models were selected by the highest area under the receiver operating characteristic curve among a limited prespecified set of candidate pipelines and were evaluated using Fast Correlation-Based Filter feature selection nested within each leave-one-out cross-validation training fold. Results should be interpreted as hypothesis-generating, particularly for endpoints with few events; Table S4: Distribution of first biopsy-proven rejection episodes by post-transplant time interval and recorded rejection phenotype.

## Abbreviations

The following abbreviations are used in this manuscript:

ABMR	antibody-mediated rejection
AUC	area under the receiver operating characteristic curve
CI	confidence interval
C-index	concordance index
DCA	decision curve analysis
DSA	donor-specific anti-HLA antibody
FCBF	Fast Correlation-Based Filter
FTIR	Fourier-transform infrared spectroscopy
HIV	human immunodeficiency virus
HLA	human leukocyte antigen
HR	hazard ratio
IQR	interquartile range
LOOCV	leave-one-out cross-validation
mRNA	messenger RNA
PRA-CDC	panel-reactive antibody measured by complement-dependent cytotoxicity
SD	standard deviation
STROBE	Strengthening the Reporting of Observational Studies in Epidemiology
TCMR	T cell-mediated rejection
TRIPOD	Transparent Reporting of a multivariable prediction model for Individual Prognosis or Diagnosis
TRIPOD+AI	TRIPOD extension for prediction models using artificial intelligence
ULS	Unidade Local de Saúde
