# Supplementary material for: Evaluation of a Pre-Transplant Serum FTIR Rejection-Risk Score Across Time Horizons and Rejection Phenotype-Defined Endpoints in Kidney Transplantation
Source: J Pers Med. 2026 Sep 21;16(9):485. doi: 10.3390/jpm16090485 (PMC13608802; doi:10.3390/jpm16090485)
Supplement: Supplementary file 1 [file jpm-16-00485-s001.zip › jpm-4521108-supplementary.pdf]

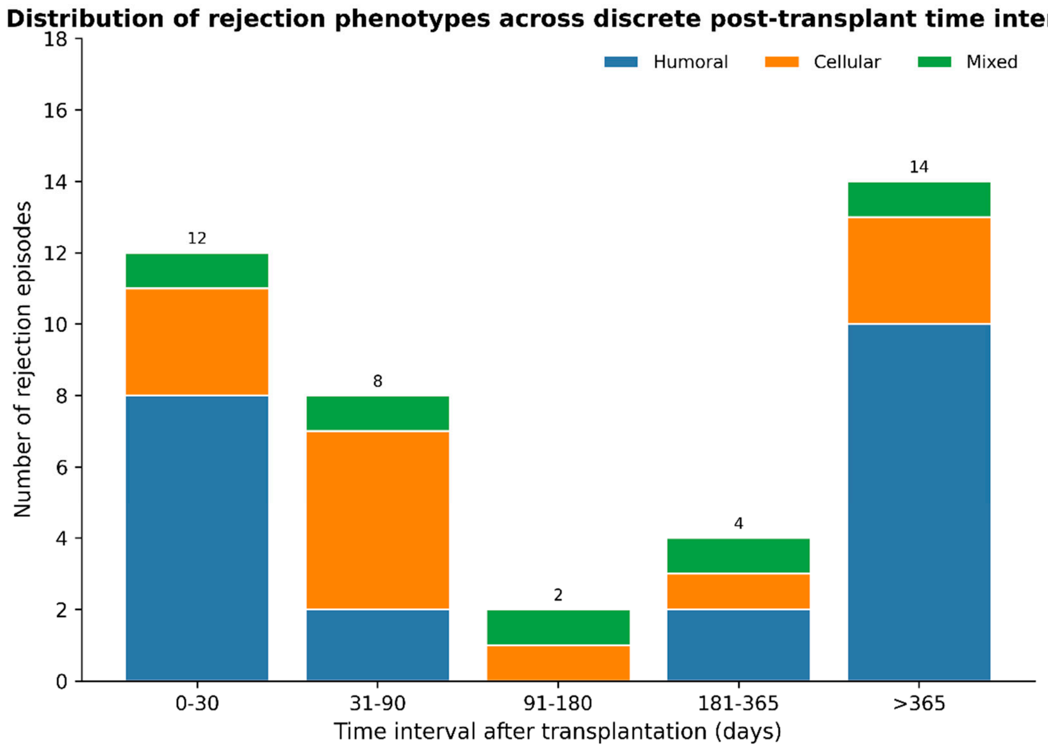

Figure S1. Distribution of rejection immunophenotypes across discrete post-transplant time intervals. The number of humoral, cellular, and mixed rejection episodes occurring within 0–30, 31–90, 91–180, 181–365, and >365 days after transplantation is shown.

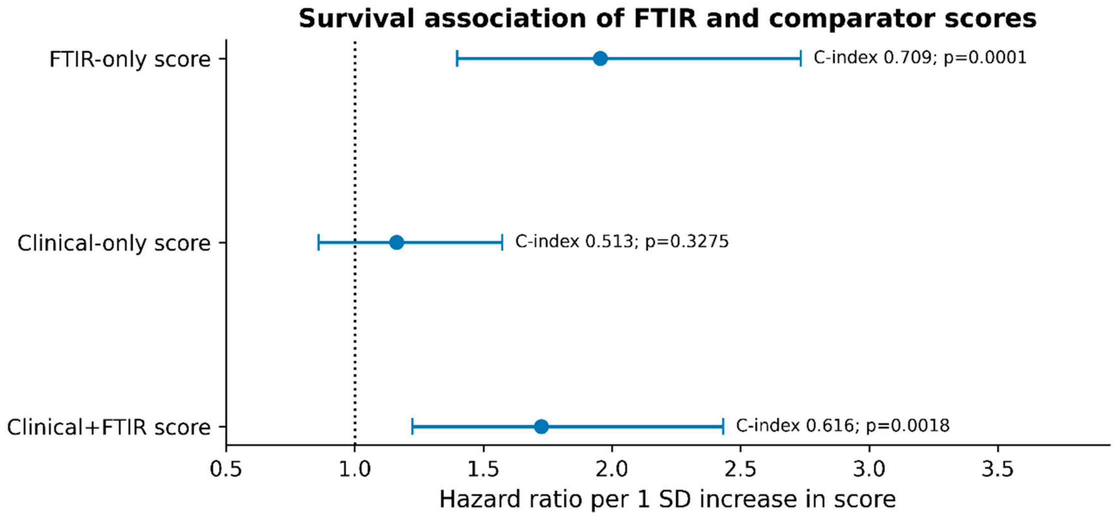

Figure S2. Comparison of the associations between the evaluated scores and biopsy-proven rejection. Hazard ratios per one standard deviation increase and corresponding 95% confidence intervals are shown for the FTIR-only, clinical-only, and clinical plus FTIRS scores. C-index values are additionally reported as measures of time-to-event discrimination.

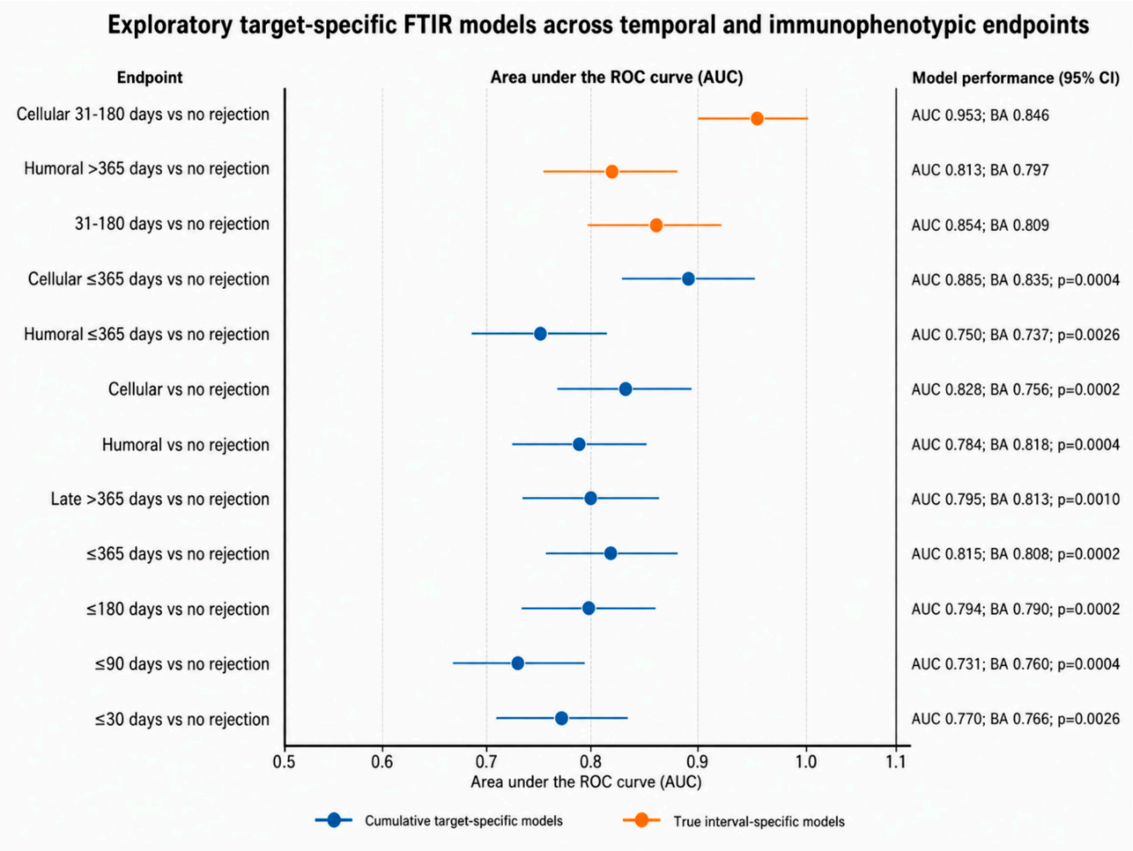

Figure S3. Exploratory target-specific FTIR model performance across temporal and rejection-phenotype-defined endpoints. Points represent the area under the receiver operating characteristic curve. Blue horizontal lines represent bootstrap 95% confidence intervals available for the cumulative and rejection-phenotype-specific models. Orange points represent selected discrete interval-specific models; no confidence intervals are reported for these models because the corresponding bootstrap estimates were not available in the analytical output. Adjacent labels report balanced accuracy and, where available, permutation-screening p-values. These analyses were secondary and hypothesis-generating.

Table S1. Performance of exploratory temporal and rejection-phenotype-specific FTIRS models selected by balanced accuracy.

Endpoint	n (events/ controls)	Preprocessing	Classifier	AUC (95% CI)	Accuracy	Sensitivity	Specificity	Balanced accuracy	F1 score	Median selected features
**Selected temporal endpoints**										
Rejection ≤30 days	51 (12/39)	Rubber-band correction + vector normalization	KNN (k = 5; distance weighting)	0.770 (0.626–0.927)	0.863	0.583	0.949	0.766	0.667	1
Rejection ≤90 days	59 (20/39)	First derivative	Linear SVM (balanced; C = 1)	0.731 (0.562–0.891)	0.780	0.700	0.821	0.760	0.683	2
Rejection ≤180 days	61 (22/39)	First derivative	RBF-SVM (balanced; C = 1)	0.794 (0.647–0.916)	0.820	0.682	0.897	0.790	0.732	2
Rejection ≤365 days	65 (26/39)	First derivative	Logistic regression (balanced; C = 1)	0.815 (0.703–0.925)	0.815	0.769	0.846	0.808	0.769	2
Late rejection >365 days	53 (14/39)	Rubber-band correction + vector normalization	Linear SVM (balanced; C = 0.1)	0.795 (0.603–0.947)	0.792	0.857	0.769	0.813	0.686	1
**Rejection-phenotype endpoints**										
Humoral rejection ≤365 days	51 (12/39)	Second derivative + normalization	KNN (k = 5; distance weighting)	0.750 (0.580–0.914)	0.863	0.500	0.974	0.737	0.632	3
Humoral rejection, any time	61 (22/39)	Rubber-band correction + vector normalization	RBF-SVM (balanced; C = 1)	0.784 (0.629–0.907)	0.869	0.636	1.000	0.818	0.778	1
Cellular rejection ≤365 days	49 (10/39)	First derivative	Linear SVM (balanced; C = 0.1)	0.885 (0.741–0.977)	0.796	0.900	0.769	0.835	0.643	1
Cellular rejection, any time	52 (13/39)	Second derivative + normalization	Linear SVM (balanced; C = 1)	0.828 (0.652–0.958)	0.827	0.615	0.897	0.756	0.640	6

All models used the prespecified 600–1900 and 2800–3400 cm^{-1} spectral windows. Fast Correlation-Based Filter feature selection was nested within each leave-one-out cross-validation training fold. Controls had five-year follow-up without biopsy-proven rejection. These analyses were secondary and hypothesis-generating. AUC, area under the receiver operating characteristic curve; CI, confidence interval; F1, F1 score; FCBF, Fast Correlation-Based Filter; FTIR, Fourier-transform infrared spectroscopy; KNN, k-nearest neighbours; LOOCV, leave-one-out cross-validation; RBF-SVM, radial basis function support vector machine; SVM, support vector machine.

Table S2. Permutation-based screening of selected out-of-fold scores from exploratory target-specific FTIR models. These analyses assessed whether the observed discrimination of the selected out-of-fold scores exceeded that expected after random reassignment of endpoint labels; they did not constitute complete model-retraining permutation tests.

Endpoint	n (events/controls)	Preprocessing	Classifier	Observed AUC	Permutations with AUC ≥ observed (n/5000)	Empirical p-value
Rejection ≤30 days	51 (12/39)	Rubber-band + vector normalization	KNN (k = 5; distance weighting)	0.770	12/5000	0.0026
Rejection ≤90 days	59 (20/39)	First derivative	Linear SVM (balanced; C = 1)	0.731	1/5000	0.0004
Rejection ≤180 days	61 (22/39)	First derivative	RBF-SVM (balanced; C = 1)	0.794	0/5000	0.0002
Rejection ≤365 days	65 (26/39)	First derivative	Logistic regression (balanced; C = 1)	0.815	0/5000	0.0002
Late rejection >365 days	53 (14/39)	Rubber-band + vector normalization	Linear SVM (balanced; C = 0.1)	0.795	4/5000	0.0010
Humoral rejection ≤365 days	51 (12/39)	Second derivative + normalization	KNN (k = 5; distance weighting)	0.750	12/5000	0.0026
Humoral rejection, any time	61 (22/39)	Rubber-band + vector normalization	RBF-SVM (balanced; C = 1)	0.784	1/5000	0.0004
Cellular rejection ≤365 days	49 (10/39)	First derivative	Linear SVM (balanced; C = 0.1)	0.885	1/5000	0.0004
Cellular rejection, any time	52 (13/39)	Second derivative + normalization	Linear SVM (balanced; C = 1)	0.828	0/5000	0.0002

All endpoints were compared with 39 recipients with five-year follow-up without biopsy-proven rejection. For each endpoint, rejection labels were permuted 5000 times while the selected out-of-fold scores were held fixed. The complete preprocessing, feature-selection, classifier-selection, and model-fitting pipeline was not repeated within each permutation; therefore, these analyses represent score-level permutation screening rather than full model-development permutation tests. Empirical p-values were calculated as (k + 1)/(5000 + 1), where k is the number of permuted AUCs greater than or equal to the observed AUC. AUC, area under the receiver operating characteristic curve; FTIR, Fourier-transform infrared spectroscopy; KNN, k-nearest neighbours; RBF-SVM, radial basis function support vector machine; SVM, support vector machine.

Table S3. Best-performing exploratory FTIRS models for discrete rejection-time intervals. Models were selected by the highest area under the receiver operating characteristic curve among a limited prespecified set of candidate pipelines and were evaluated using Fast Correlation-Based Filter feature selection nested within each leave-one-out cross-validation training fold. Results should be interpreted as hypothesis-generating, particularly for endpoints with few events.

Endpoint	n (events/controls)	Preprocessing	Classifier	AUC	Accuracy	Sensitivity	Specificity	Balanced accuracy	F1 score	TN/FP/FN/TP	Median selected features
Rejection 0–30 days vs 5-year rejection-free controls	51 (12/39)	First derivative + normalization	Naïve Bayes	0.761	0.765	0.417	0.872	0.644	0.455	34/5/7/5	1
Humoral rejection 0–30 days vs 5-year rejection-free controls	47 (8/39)	First derivative	Logistic regression (C = 0.1)	0.801	0.702	0.500	0.744	0.622	0.364	29/10/4/4	2
Rejection 31–90 days vs 5-year rejection-free controls	47 (8/39)	First derivative	Logistic regression (C = 0.1)	0.788	0.617	0.875	0.564	0.720	0.438	22/17/1/7	1
Rejection 31–180 days vs 5-year rejection-free controls	49 (10/39)	First derivative	Logistic regression (C = 0.1)	0.854	0.755	0.900	0.718	0.809	0.600	28/11/1/9	1
Cellular rejection 31–180 days vs 5-year rejection-free controls	45 (6/39)	First derivative	Logistic regression (C = 0.1)	0.953	0.733	1.000	0.692	0.846	0.500	27/12/0/6	1
Rejection >365 days vs 5-year rejection-free controls	53 (14/39)	First derivative	Logistic regression (C = 0.1)	0.839	0.736	0.714	0.744	0.729	0.588	29/10/4/10	3
Humoral rejection >365 days vs 5-year rejection-free controls	49 (10/39)	Second derivative + normalization	Logistic regression (C = 0.1)	0.813	0.796	0.800	0.795	0.797	0.615	31/8/2/8	3

All models used the prespecified 600–1900 and 2800–3400 cm^{-1} spectral windows. Controls had five-year follow-up without biopsy-proven rejection. AUC, area under the receiver operating characteristic curve; F1, F1 score; FCBF, Fast Correlation-Based Filter; FN, false negative; FP, false positive; FTIRS, Fourier-transform infrared spectroscopy; LOOCV, leave-one-out cross-validation; TN, true negative; TP, true positive

Table S4. Distribution of first biopsy-proven rejection episodes by post-transplant time interval and recorded rejection phenotype.

Time interval	All rejection episodes	Humoral	Cellular	Mixed
0–30 days	12	8	3	1
31–90 days	8	2	5	1
91–180 days	2	0	1	1
181–365 days	4	2	1	1
>365 days	14	10	3	1
**Total**	**40**	**22**	**13**	**5**
